# An analysis of migration and implications for health in government policy of South Africa

**DOI:** 10.1186/s12939-023-01862-1

**Published:** 2023-05-08

**Authors:** Karima Manji, Shehani Perera, Johanna Hanefeld, Jo Vearey, Jill Olivier, Lucy Gilson, Helen Walls

**Affiliations:** 1 London School of Hygiene & Tropical Medicine (LSHTM), Faculty of Public Health and Policy, Department of Global Health and Development, London, UK; 2 School of Public Health and Family Medicine, Faculty of Health Sciences, University of Cape Town, Cape Town, South Africa; 3 The African Centre for Migration & Society (ACMS), University of the Witwatersrand, Johannesburg, South Africa

**Keywords:** Migration, Migrant, Universal health coverage, Health policy, Health system, South Africa

## Abstract

For over a decade, the global health community has advanced policy engagement with migration and health, as reflected in multiple global-led initiatives. These initiatives have called on governments to provide universal health coverage to all people, regardless of their migratory and/or legal status. South Africa is a middle-income country that experiences high levels of cross-border and internal migration, with the right to health enshrined in its Constitution. A National Health Insurance Bill also commits the South African public health system to universal health coverage, including for migrant and mobile groups. We conducted a study of government policy documents (from the health sector and other sectors) that in our view should be relevant to issues of migration and health, at national and subnational levels in South Africa. We did so to explore how migration is framed by key government decision makers, and to understand whether positions present in the documents support a migrant-aware and migrant-inclusive approach, in line with South Africa’s policy commitments. This study was conducted between 2019 and 2021, and included analysis of 227 documents, from 2002–2019. Fewer than half the documents identified (101) engaged directly with migration as an issue, indicating a lack of prioritisation in the policy discourse. Across these documents, we found that the language or discourse across government levels and sectors focused mainly on the potential negative aspects of migration, including in policies that explicitly refer to health. The discourse often emphasised the prevalence of cross-border migration and diseases, the relationship between immigration and security risks, and the burden of migration on health systems and other government resources. These positions attribute blame to migrant groups, potentially fuelling nationalist and anti-migrant sentiment and largely obscuring the issue of internal mobility, all of which could also undermine the constructive engagement necessary to support effective responses to migration and health. We provide suggestions on how to advance engagement with issues of migration and health in order for South Africa and countries of a similar context in regard to migration to meet the goal of inclusion and equity for migrant and mobile groups.

**Supplementary Information:**

The online version contains supplementary material available at 10.1186/s12939-023-01862-1.

## Background

Migration and health are increasingly recognised as a global public health priority [1, 2]. Key global-level initiatives that promote and facilitate engagement with this issue include, the 2008 World Health Assembly (WHA) Resolution 61.17 on the health of migrants [3], the 2017 WHA Resolution 70.15 promoting the health of refugees and migrants [4], which led to the development of the global action plan of the World Health Organisation’s (WHO) to promote the health of refugees and migrants (2019–2023) [5], and the global consultations on migration and health in 2010 and 2017 [6, 7]. These initiatives call on member states to improve responses to the health of migrants, including the response of health systems to migration. In this spirit, the 2018 Lancet Commission on Migration [8] emphasises the responsibility of governments to uphold human rights and provide equitable universal health coverage (UHC) to migrant populations, regardless of their legal status. This goal is also reflected in the 2019 WHA agreement [9]: that a key WHO member state priority is to integrate migration and refugee health into health policies.

Within these migration and health policy frameworks, and indeed over the course of almost two decades, the framing of health by the public health community has increasingly focused on the social determinants of health [10] – or the factors other than health care that are influenced by social policies and shape health in powerful ways [11]. Similarly today, the issue of migrants’ urban living and working conditions, and links to (poor) health are well documented in the literature [12–14]. For example, some groups of migrants, including undocumented migrants – international migrants without legal documentation – may be more vulnerable to poor health outcomes, not only due to restrictive policies on health access, but also due to unsafe urban working and living conditions [1]. Indeed, there is increasing scholarship underscoring the relationship between migration and the social determinants of health [2, 15], including global recognition that social and development agendas, and approaches to the management of (im)migration are key determinants of migrant health [16–18].

Concerns about the ways in which health is increasingly co-opted by approaches to national security and restrictions on movement are well documented by the public health and migration community [19–22], and highlight the importance of bringing together the governance of health and immigration to address the health of migrants. Immigration policy agendas that focus on security concerns linked to migration are often considered by the international public health community to be damaging and xenophobic, impairing the health of migrants by excluding, discriminating and/or blaming migrants as vectors of diseases [16]. Furthermore, increasingly restrictive immigration policies can drive people to cross international borders in irregular and unsafe ways, undermining opportunities to advance a migrant-inclusive health agenda [23]. Migration is a determinant of health; although some migrants do face distinct vulnerabilities to poor health, these are exacerbated by migrant-unfriendly or migrant-indifferent legal and policy frameworks and health systems, and resolving these issues will require intersectoral approaches [2].

The Sustainable Development Goals further underscore the relationship between health, migration and development, and identify as a key target coordinated responses between multiple sectors as a means to improve responses to migration and health [2, 8]. Effective solutions entail migration-aware approaches including within (but not only) the health sector [23]. Or put alternatively, as migration and health is a cross-cutting issue relating to (but not limited to) sectors addressing development, trade, labour, housing, finance and security, policy coherence across sectors is needed to support effective responses. Linking the health of migrants to broader agendas focused on addressing social inequalities and strengthening health systems that are responsive to diversity and population mobility supports the successful integration of mobile populations into heath care systems and the achievement of UHC, while simultaneously challenging populist xenophobic rhetoric that undermines such achievement [23].

Despite efforts by global health actors to advance the migration and health agenda to achieve health for all, providing UHC is a major undertaking and challenges persist [2, 24]. Health systems face multiple priorities challenging the achievement of UHC that vary across contexts. It has been noted, however, that across country settings of all income levels, far more policy attention is devoted to cross-border migration – the movement of people across international borders, including asylum seekers, refugees, migrant workers and undocumented migrants – than to internal migration – the often more prevalent movements of people within their country of birth [24, 25]. While cross-border migrants, particularly those who are undocumented (defined earlier) face particular challenges in accessing healthcare, many of these difficulties are experienced by both cross-border and internal migrants [24].

South Africa (SA), a middle-income country, experiences high levels of different types of migration, both cross-border and internal migration [26–28]. In 1996, soon after the fall of the Apartheid government, SA established the ‘Right to Health’ through its Constitution [29] and since this time has worked to build its public system to one that promotes health equity in light of the apartheid-era inequities [30, 31]. However, the persistently large public–private split in SA’s health system – with a powerful private health sector servicing a small proportion of the wealthier population – poses an important enduring challenge to achieving UHC. For instance, there are deficiencies in the public health system as a result of the unequal distribution of resources between the well-funded private sector over the poorly-resourced public health sector [32–34]. The proposed system of National Health Insurance (NHI) [35] seeks to address this gap, including through commitment to inclusivity and the care for migrant and mobile groups. At the same time, an important wider context for the issue of addressing migration and health in SA is the persistence of high levels of xenophobia and waves of violence resulting from these attitudes and stereotypes that many migrant groups face in the country [36, 37].

As researchers with a work programme examining key gaps in evidence on migration and their impacts on health systems in SA [24, 38, 39], we are interested in understanding whether the South African government is advancing a ‘migrant-inclusive and migration-aware approach to UHC’ (p.248) [23]. Institutional policies, including those of government, have been conceptualised by sociology scholars as idealised schemata for establishing social order and shaping reality in a certain way while offering (governance) mechanisms for problem-solving [40]. Policy texts constitute a major data source for analysing government and other institutional intentions, revealing how policy issues are defined, how the causes of issues addressed on political agendas are understood, and the nature of proposed solutions [41, 42]. Analyses of policy texts have become increasingly common in the field of health policy analysis in recent years, given their potential to illuminate the nature of policy debates and help researchers to understand responses to complex policy issues [43].

We undertook this study to systematically examine whether published policies of the South African government engage with migration in relation to health and the wider social and structural determinants of health, how this issue is described and framed by these key decision makers, and if any and what types of policy solutions are proposed. We frame migration to include cross-border movement (inclusive of refugees, asylum seekers, undocumented migrants and migrant workers), and internal migration (the movement of people within SA). This study was undertaken to inform debate within the public health and migration community about whether or not migration-aware and migration-inclusive approaches exist in SA, to facilitate UHC for all, regardless of migratory and/or legal status – and to support the development of improved policy responses.

## Methods

We undertook this analysis of South African government policy documents (101 in total, from a possible 227 identified documents) between 2019 and 2021. Given the multi-sectoral nature of the issue of migration and health [2], our analysis includes: 1) policies that refer to migration and health explicitly and; 2) policies that refer to migration in relation to the social and structural determinants of health, but do not mention health explicitly.

Our analysis covers a wide policy spectrum, and also different levels of government in SA (national, provincial and metropolitan levels). The decision to include policies at both national and sub-national levels in SA was taken for several reasons. Firstly, it reflects views from prominent scholars on migration in SA that the consequences of migration, both positive and negative, may be most acute at the municipality levels, where the impact of migration on local level services, and vice versa, is tangible [44]; and that engaging with policy experiences at all levels of government is critical for developing a more accurate and complete understanding of policies addressing the issue of migration [45]. Secondly, this position mirrors the framing of health by the public health community and the global migration and health policy frameworks, which emphasise that the health of individuals is influenced by social policies (beyond health) that impact on people’s living and working conditions, including in the domains of housing, electricity, water, transport and the workplace. Indeed, there is scholarship from across different country and regional contexts emphasising the significance of the spatial and social determinants of urban health [46–48]. In SA, policies addressing urbanisation and the structural determinants of urban health are found mainly in the domain of the local level of government. Furthermore, given the scope of our study, it was a logical decision to explore policies linked to urbanisation to understand the dynamics of internal migration in SA, given that SA is one of the most urbanised countries in the region, with over half of the population reported to be urban [45, 49]. There are three categories of municipality in SA, focused on growing local economies and providing infrastructure and services: 278 municipalities, comprising eight metropolitan, 44 district and 226 local municipalities [50]. We studied policies relating to the eight metropolitan municipalities or ‘cities’ in SA, which include: Buffalo City (East London), the City of Cape Town, Ekurhuleni Metropolitan Municipality (East Rand), the City of eThekwini (Durban), the City of Johannesburg, Mangaung Municipality (Bloemfontein), Nelson Mandela Metropolitan Municipality (Port Elizabeth) and the city of Tshwane (Pretoria).

The main stages of the analysis included: 1) identifying policy documents with relevance to migration and health, at national and sub-national levels in SA; 2) extracting relevant data from the documents; and 3) developing a coding framework and undertaking a thematic content analysis of the extracted data. We synthesised our findings according to the key themes identified.

### Search strategy and document selection

We employed both an automated and manual search strategy to retrieve the relevant policy documents. With the automated strategy, we used key words to generate three sets of policy documents at each level of government; one set linked to migration-specific documents; the second set linked to health-specific documents; and the third set of policy documents at the intersection of migration and health (see Additional file 1). For this purpose, we searched Google Scholar and Scopus, local and national government websites and databases, and the University of Cape Town Libraries’ policy databases. We also retrieved governmental strategic reports, financial reports, quarterly reports and annual performance plans. We expected to identify only a small number of documents addressing issues of migration, and for reasons including that SA does not have a government department dedicated to issues of migration. To identify additional policy documents engaging with migration, in relation to health, that did not show up in our automated search, we manually located and screened policies in the health sector and other governmental departments relevant to the scope of our study. As well as searching the department of (im)migration governance and security, we searched additional sectors at the intersection of migration and health, including, development, labour, housing, transport, trade and industry, and environmental affairs, and retrieved policy documents across these sectors. We subsequently screened the policies retrieved from both the automated and manual searches, for reference to migration and health explicitly (in the health policies), and any mention of migration in the non-health policies. We completed our manual search once we reached data saturation i.e. when no new themes linked to migration and health were identified in subsequent documents.

We originally discussed including documents from 2007/2008 to 2019 (2019 being the year when the search was undertaken), with the lower limit intended to reflect the build-up to the general elections in 2009 that resulted in a new provincial legislative administration, and which may have impacted on policy relevant to migration and health. However, given that national processes do not always reflect local ones, and vice versa, and that we found few policies addressing migration and health-related issues during this specified period at the metropolitan municipality level, we pushed back the time frame to 2002 in our search for policies at the local level, while applying the original lower limit (2007/2008) to our search at the national and provincial levels. We took this decision to support our analysis and ensure we had not missed any documents that engage with migration and health-related issues at the local level.

### Data extraction

To capture and collate the data from across the documents, we developed a standard coding template, with categories that included: 1) the year of each policy document; 2) the specific terms used to describe migration, to distinguish internal and cross-border migration, and to denote, where described, the group of migrants in question (including whether a distinction is made between undocumented migrants, refugees, asylum seekers and/or migrant workers); 3) the wording of migration issues—whether the issues are described as an opportunity or a problem, including the associated views; and 4) any specific recommendations for stakeholder groups, including the public and/or the health sector. We extracted these data, where available, from each document and entered them into the coding template, developed in Excel (see Additional file 2).

### Data analysis

All the study co-authors contributed to the development of codes for data categorisation, based on an initial review and discussion of the data sets. Following this, we (KM and SP) independently applied the coding framework across the data sets and expanded the framework, to include any new codes identified, in the subsequent policies included in our analysis. All the researchers discussed any discrepancies in coding the data. Each discrepancy was a case of one reviewer applying one of the original codes developed to label the data, with the other reviewer categorising the data under a new (but related) code. As the data analysis evolved and we identified common patterns across data sets, it became apparent that all the newly developed codes converged under the original codes we developed. For example, the sub-codes ‘border management’ and ‘legality’ were identified across several policies and converged under the label ‘national security’, which covered a range of concerns linked to cross-border migration, including border control and security issues.

As an additional layer of cross- checking the reliability of the codes applied across the data sets, two of the researchers (JV and HW) retrieved and screened 10% of the documents, selected at random, from the full set of documents included in the analysis. The additional codes identified by the researchers were applied across the entire data set, to further develop the themes and analysis. To synthesise the thematic analysis, KM identified associations and patterns within and across the coded data that we then developed into overarching themes, which represent the narrative underpinning the study findings. All researchers were involved in the interpretation of the study findings.

## Findings

### Migration is a neglected policy issue

The issue of migration is covered to different extents by the government policy at different levels (Table 1). Just over half the policies the national level and less than half the policies at the sub-national levels addressed migration to a degree that make it possible to analyse how the issue is positioned. With the remaining policies reviewed, we identified two categories: 1) those policies that made no reference to the issue and; 2) those that mentioned migration but to a small extent that did not enable coding of the data into any meaningful categories.

**Table 1:** Coverage of migration and health issues across the policy discourse

	**Policies offering scope for analysis**	**Policies with negligible or no mention of issue**	**Total**
**National**	34 (53%)	30 (47%)	64
**Provincial**	33 (45%)	40 (55%)	73
**Metropolitan Municipality**	34 (38%)	56 (62%)	90
**Total**	101	126	227

With regard to the 101 policies identified across the different levels that did offer us the scope to conduct a thematic analysis, and which were included in our study (see Additional file 3) the majority of them engaged with migration and health-related issues in a limited way. For example, we found only one policy document, at the national level, within the time-frame specified (2007/2008–2019) that was migration-specific (The White Paper on International Migration, 2017). In addition, many of the policies included in the analysis demonstrated only limited engagement with the issue of migration, and did not contain enough data to cover all the data extraction categories described earlier. Further, whereas most (but not all) of the documents defined types of population mobility i.e. whether specific to internal migration and/or cross-border movement generally (see Table 2 below), they rarely specified, in the case of cross-border migrants, the specific migrant group i.e. whether undocumented migrants, refugees and/or asylum seekers. These findings reveal the lack of attention paid to migration in relation to health, in the South African policy discourse.

**Table 2:** Key themes in relation to migration and health in the policy documentation at each level of government

	**Salient themes**						
	**Migration associated with diseases of concern**	**Efforts needed to mitigate health threats from migration**	**Cross-border mobility as a threat to national security**	**Migration linked to population growth and/or urbanisation**	**Local sectors’ engagement with migration**	**Labour migration as a concern**	**Health worker mobility**
**National level**	**✓**	**✓**	**✓**			**✓**	**✓**
**Provincial level**				**✓**			**✓**
**Metropolitan level**			**✓**	**✓**	**✓**		
	**Type of population mobility associated with each theme**						
**National level**	-Urbanisation (NCDs*) -Migrant workers, travelers, seasonal farm workers and refugees moving across borders (malaria) -illegal migrants (malaria) -Travelers returning from endemic countries (malaria) -Mobile populations, migrants, undocumented foreigners, labour migrants (HIV, TB & STIs)	-Urban migrants (NCDs*) -Migrant populations (TB) —Migrant workers, travelers and seasonal farm workers moving across borders (malaria) -Recreational travelers and migrant labourers (malaria) -Cross-border migrant labourers (TB)	-Migration of foreign nationals -Immigration -Migration at ports of entry			-Outward labour migration -Iinternational migrants; skilled and business persons -Artisan immigrants -Inflow of people with priority skills -Inflow of skilled labour	-Foreign health workers
**Provincial level**				-Migration & urbanisation -Cross-border mobility -Migration into provinces -Inter-provincial migration			-Foreign health workers
**Metropolitan level**			-Illegal migration -Irregular migration -Illegal migrants	-Immigration towards urban areas -Intra-urban migration -Families settling in provincial cities -Migration into urban areas -Rapid urbanisation -In-migration -Immigration & urbanisation -Urbanisation & migration -Urban migrants -Circulatory migration & rapid urbanisation	-Urban migrants & newcomers -Urban migrants -City’s visitors -Rural–urban migration		

In the policies containing sufficient material for an analysis of key themes, we identified seven key themes, summarised in Table 2. These themes reflect how issues of migration in relation to health and the social determinants of health are commonly presented within and across the national and sub-national government policy discourse in SA.

### Migration associated with diseases of concern

Most policies at the intersection of migration and health at national level focus on migrant and mobile groups’ vulnerability to diseases of concern, including non-communicable diseases (NCDs) and (the spread of) communicable diseases.

The Strategy for Prevention and Control of Obesity in South Africa (2015–2020), the Breast Cancer Prevention and Control Policy (2017) and The Cervical Cancer Prevention and Control Policy (2017) consider migrants as a ‘high-risk group’ and associate increasing urbanisation—particularly, lifestyle changes linked to the urban environment—with an increased risk of NCDs among urban migrants.

The National Department of Health (NDoH) policies addressing malaria focus mainly on migration in terms of the association of malaria with cross-border mobility, attributing the spread of malaria to specific ‘high-risk’ migrant groups. In both the Malaria Elimination Strategic Plans (2012–2018 and 2019–2023) identified risk groups include migrant workers, travelers, seasonal farm workers and refugees moving through malaria endemic areas within SA and across borders. In these policies, as in the National Guidelines for the Prevention of Malaria (2018), malaria endemic areas are identified as those bordering other countries. Other language in the 2012–2018 policy conveys a sense of blame for the spread of malaria on migrant groups who come to the country illegitimately, through descriptions of infections stemming from ‘illegal immigrants’ and ‘porous borders’, and the view that ‘importation of cases from African countries remains a threat’.

The notion that cross-border migrants are associated with a high-risk of disease transmission is mirrored in SA’s National Strategic Plan for human immunodeficiency virus (HIV), tuberculosis (TB) and sexually transmitted infections (STIs) (2017–2022). This policy conceptualises mobile populations, migrants and undocumented foreigners as ‘vulnerable for HIV and STIs’. In this policy ‘labour migrants, specifically those involved in big construction and infrastructure projects’ are specifically mentioned in this regard.

### Improving the health system and boosting collaborations with neighbouring countries to mitigate threats from migration

Multiple policies at national level advocate improving the health system to address the perceived health threats posed by migration. The Strategy for the Prevention and Control of Obesity in SA (2015–2020) pushes to ‘increase knowledge among [the high-risk urban migrant] population and implement a multi-faceted effort to prevent obesity’. The idea of strengthening health systems to tackle the issues resulting from migration is also reflected in policies addressing communicable diseases. The TB Strategic Plan for SA (2007–2011) seeks to ‘determine the barriers they [the poor and vulnerable groups, migrant populations, the homeless and ethnic minorities] face in accessing services and develop interventions to address these [barriers]. In addition, both the Malaria Elimination Strategic Plans (2012–2018 and 2019–2023) state that to mitigate the threats posed by specific groups of migrants [migrant workers, travelers and seasonal farm workers moving across borders], case detection and management efforts will be required at the primary health care level to avoid secondary transmission and that ‘messages should be disseminated throughout the year, with intensification leading up to the malaria season and around peak holiday seasons when there is significant population movement in SA and across borders’. The 2019–2023 document additionally specifies that ‘health promotion and communication for malaria elimination activities should be specifically directed at recreational travelers or migrant labourers and will need to be disseminated at border posts, taxis, bus stations and work places’. As seen across these policies, the solutions posed to strengthen health systems to address the health threats perceived as a result of migration are focused on individual-level approaches, targeting specific ‘high-risk’ migrant groups.

Another recurring theme in national level health policies, to mitigate perceived threats from migration, is the need to bolster collaborations with neighbouring countries, where the threat of communicable diseases is seen to arise. Both the Malaria Elimination Strategic Plans (2012–2018 and 2019–2023) state that ‘given the high rate of imported malaria in SA, collaboration with neighbouring malaria endemic countries is fundamental to reducing importation risk and preventing local transmission’. Similarly, the National Strategic Plan for HIV, TB and STIs (2017) emphasises that communicable disease risk is a cross-border issue and the government must act to ‘increase cross-border cooperation to improve responses for migrant labourers, especially with regards to TB, by strengthening cross-border cooperation with neighbouring countries and other stakeholders’. Both the above positions are examples of the perception at national level that cross-border migration is problematic, with the risk of local transmission attributed to cross-border migrant groups.

### Labour migration as a concern for national development

The issue of labour migration is exclusively addressed at national level where it is repeatedly described as an important factor for SA’s development. In particular, several policies articulate the negative impact of labour migration on development. The National Development Plan (2030) entitled ‘Our Future Make It Work’, of the Office of the Presidency, denotes ‘outward labour migration as a significant risk to development’ and links the migration of skilled people from developing countries to the current economic slowdown in these countries.

In response to the issue of unfavourable labour migration, multiple efforts are outlined by national government to support the implementation of immigration strategies that make provisions for coveted groups of international labour migrants. The White Paper on International Migration (2017) acknowledges that ‘migration can promote economic growth in SA, if the country grants [specifically] business, critical skills, study and visitors’ visas’. This policy (only) makes provision for economically established and highly-skilled migrants, stating that ‘SA has not been successful in attracting and retaining sought-after international migrants, such as skilled and business persons’. The Joint Initiative on Priority Skills Acquisition (2008) of the Office of the Presidency describes the scarce skills quota list published by the Department of Home Affairs (DHA) to support its plan of enabling specific groups of immigrants to enter the country to source work as artisans in scarce trades linked to construction. The Human Resource Development Strategy for South Africa (2010–2013) of the Department of Education highlights the shortcomings of immigration quota lists, claiming that they ‘confound the demands of the labour market, failing to result in a net positive inflow of people with priority skills required for economic growth and development’. In particular, the document articulates as a strategic priority, a push to increase the number of skilled personnel in the areas of design, engineering and artisan trades. Following suite, The New Growth Path Framework recommends that ‘the overall supply of highly skilled labour should be increased by continued efforts to streamline the immigration system in ways conducive to the inflow of skills’, while also advocating ‘the ongoing commitment to upgrade domestic education on a broad basis, to meet labour demands’.

### Population growth linked to migration placing pressure on the health system

In the health sector at provincial level, population growth in cities linked to migration is interpreted as problematic in terms of health service provision. The Gauteng DoH Annual Report (2013–2014) indicates that ‘cross-border utilisation of health services and high rates of migration into the provinces increases the size of the population, with a negative impact on service delivery’. A subsequent Gauteng DoH Annual Report (2017–2018) describes the ‘increasing demand of out-patient services resulting from rapid urbanisation’. We identified multiple Annual Reports in the health sector pertaining to Mpumalanga, (from 2014 through till 2019) which emphasise that ‘migration from shared borders between Limpopo, Gauteng, Free State, KwaZulu-Natal and Mpumalanga poses a challenge in rendering health services, as demands can never be projected accurately in terms of planning and resource allocation’. Policies pertaining to the other provinces also highlight the link between urbanisation and the increasing demand for health services. The KwaZulu-Natal DoH Annual Performance Plan (2012–2013) attributes population growth in informal settlements in economic hubs to urbanisation, which is said to put ‘additional unforeseen pressure on service delivery’. Similarly, the Eastern Cape DoH Annual Report (2015–2016) also describes ‘rural to urban migration linked to economic factors and the resulting mushrooming of informal settlements as leading to a high demand for health services’.

### Migration in the context of urbanisation placing pressure on public services

Similarly to at provincial level, the link between migration and population growth, in the context of urbanisation, is reiterated across policies at sub-national level. Furthermore, urban growth linked to migration is also seen as exacerbating the strain on local public services and amenities.

The Metropolitan Spatial Development Framework of Mangaung (2020) emphasises that ‘migration into urban areas increases pressure on services and transport’. In a similar light, the document entitled Provision of Road Tolls in the City of Cape Town (2004) states that ‘owing to the severe financial constraints and the pressures of rapid urbanisation [and] population growth […], the city has been struggling […] in respect of the transport system, including the provision of roads and maintenance thereof for the past two decades’. Concerns are also raised about the implications of population growth in cities with regards to water provision. The Ekurhuleni Environmental Policy and Implementation Plan (2013) points to the ‘rate of urbanisation and population growth as degrading and threatening the sustainability of land and water resources’. Similarly, Cape Town’s Climate Change Policy (2017) highlights that the ‘city’s water supplies are coming under threat as a result of several factors, including an increase in demand caused by population growth linked to in-migration’.

In particular, the issue of inadequate housing linked to urban growth is ubiquitous at the local level. Multiple policies pertaining to the different metropolitan municipalities illustrate the link between migration into cities and the problem of accommodation shortages and housing backlogs. The City of Tshwane’s Annual Report (2018–2019) reiterates findings from the 2012–2014 Annual Report, regarding the ‘challenges of formalising the city’s informal settlements and meeting demands for low-cost housing caused by immigration, population growth and urbanisation, which aggravates the existing challenges of addressing housing backlogs in the city’. The Integrated Development Plan (2015–2016) for eThekwini highlights that ‘migration has implications for housing and basic household services backlogs’. Similarly, Mangaung’s Annual Report (2017–2018) cites figures from a community survey to illustrate ‘the ever-growing housing backlog in the city as fueled by pressures of urbanisation, migration and population increase’. This issue is reiterated in the city’s metropolitan Spatial Development Framework (2020) that highlights accommodation challenges, including ‘financing for lower-end housing, slow progress in rental accommodation and upgrading of informal settlements’, where most urban migrants settle. The Annual Reports identified for Buffalo city (from 2011 to 2014) stress that ‘circulatory migration and rapid uncontrolled urbanisation have implications for housing tenure option types, given that some people only require temporary housing during the working week before returning to their permanent peri-urban and rural villages for the weekend’.

### Local government actors need to engage with migration

We found that the vast majority of policies that promote engagement with migration and the wider social determinants of health are those concerned specifically with the challenges and burden of population growth in cities, and they are mostly in the domain of the local government. For example, the City of Johannesburg Spatial Development Framework (2040) acknowledges that ‘the city is a main reception area for urban migrants’ and states ‘the importance of providing essential services to urban newcomers, including affordable housing, access to work and access to public transit’. This language is reflected in approaches advocated at the local level. The City of Tshwane’s Annual Report (2011–2012) recommends the provision of basic services, roads and storm water drainage to urban migrants through the equitable distribution of resources, development and implementation of the city’s long-term plan. Buffalo City’s Annual Report of 2011–2012 similarly endorses the provision of equitable and sustainable health and safety services to […] the city’s residents, citizens and visitors. The Long-term Growth and Development Plan (2017–2032) for Nelson Mandela Bay advocates the provision of sustainable urban infrastructure, including transport and adequate household structures, ‘to stimulate private investment and political engagement, for both inhabitants and newcomers’. A key finding across these policies is that although terms are used such as equity and inclusivity, there is no further description of to whom the services in question are accessible; for example, to *all* ‘newcomers’ or to ‘urban migrants’, regardless of their legal status.

In addition to proposing actions and responses to the challenges of population growth in cities, the local governments denote the role of relevant state agencies and stakeholders to address issues linked to the provision of services and amenities, in the context of migration and urbanisation. The Mangaung Metropolitan Municipality Five Year Integrated Human Settlements Plan (2016–2021) recommends engagement with the Free State Department of Public Works, Roads and Transport ‘to upgrade and maintain the roads in rural areas to improve access to urban markets and services as a way to decrease rural–urban migration’. In response to the issues of affordable and well-located housing for the ‘rapidly growing, mobile and urban population’ identified in Buffalo city (explained above), the city’s Annual Report (2013–2014) outlines its engagements with the Housing Development Agency ‘to allow for land release and acquisition’ for such housing developments. The Cape Town Bioregional Plan (2015) points to the on-going discussions between the Environmental Resource Management Department and other City Departments, State departments and developers, ‘on issues of biodiversity and competing land uses such as urban development’.

### Human resources for health in the context of health worker mobility

Several health-related policies at both national and provincial levels address the issue of health worker mobility – an issue with implications for human resources for health. Although these particular policies do not denote an explicit position linked to migration, the majority of the policies focus on the supply of foreign workers to mitigate gaps in human resources facing the health sector. The Eastern Cape DoH Strategic Plan (2012/16–2019/20) indicates that the DoH has a programme for the recruitment of qualified doctors and workers through the Foreign Health Workforce Management directorate of the NDoH, the African Health Placements for the placement of doctors at district and regional hospitals, and the NDoH Cuban Doctor Programme. The Policy Guideline on the Requirements for Practice of Medical Professionals in South Africa (2018) denotes the critical skills visa in relation to the practice of medicine in SA, including details of medical school internship eligibility, community work, and work visas linked to the practice of medicine. Similarly, the Employment of Foreign Health Professionals in the South African Health Sector (2010) document includes policy regulations for foreign health professionals seeking employment in SA.

### Cross-border mobility as a threat to national security interests

Multiple national and metropolitan level policies across different state departments link cross-border migration to security risks and advocate stricter measures to safeguard national security. Echoing sentiments in previous documents, the 2012–2013 Department of Home Affairs Strategic Plan states that ‘migration is a concern of national importance’. It stresses the agency’s need to ‘uphold the integrity of the State, to safeguard the identity and status of citizens and to regulate migration to ensure security and promote development in the country’. The White Paper on International Migration (2017), a document specific to immigration law in SA also has national sovereignty at its core:

- As a sovereign state, SA reserves the right to determine who is allowed entry into the country and under what conditions. Therefore the new White Paper on International Migration affirms SA’s sovereign right to determine the admissions and residence conditions for foreign nationals in line with its national interests.

The ‘new’ White Paper further points to the shortcomings of the first White Paper on International Migration (1999), namely the relaxation of border and immigration controls, juxtaposed with ‘the current policy of ensuring national security through the management of risks’. The paper reinforces the securitisation of migration and stricter border controls as key measures in governing migration:

- Countries with a similar risk profile of SA that effectively manage immigration, apply, to a far greater extent, the basic principle of keeping risks outside their borders. This includes doing adequate checks at missions and by airline liaison officers at key airports. The cost of these measures is far lower than dealing with threats such as fugitive crime bosses once they have established themselves in SA.

To this effect, the White paper recommends interventions that largely seek to ‘reduce irregular migration and improve compliance with immigration and related-legislation’.

We identified additional national-level policies promoting increased border control linked to migration governance. The Doing More Together Policy (2009–2014) of the Office of the Presidency and the SA Police Service Strategic Plan (2010–2014) recommend ‘the establishment of a border management agency to manage migration, customs and land borderline control services as well as the efficient coordination of services between other departments at ports of entry’.

The link between migration and security concerns is mirrored at local level of government. The Nelson Mandela Bay Integrated Development Plan (2017/18–2021/22) specifies that ‘our society is becoming a more diverse one as a result of migration over the past decade [and] community division creates tensions [and] prejudice and can lead to criminal behaviour, [with] each incident increasing the risk of many more’. Although the document does not specify the perpetrators of this criminal behaviour, it links the issue of criminal behaviour to migration, and indicates that the municipality is looking to re-establish a security agenda, in line with the government’s White Paper on Safety and Security (issued in 1994). This policy seeks to ‘improve the safety of its citizens, and prevent them from becoming victims of crime’. The explicit emphasis on citizens (versus non-citizens) as being worthy of protection reflects the view in the White papers of nationalism as a defence against security threats that come from the ‘outside’.

The association between ‘illegal migrants’ and criminality, and the need for state intervention, is reiterated across several housing-related policies pertaining to the city of Ekurhuleni, over a seven-year period. The Housing Assistance in Urgent Housing Situation Policy (2003) discusses the role of the DHA and the local police ‘with regards to addressing possible illegal immigration and the control of unruly behaviour [if] it is established that illegal immigrants [non-beneficiaries] appear to be present [in emergency housing]’. Similarly, the Procedure for the Removal of Unlawful Occupiers of Land (2005) highlights that ‘where there is a suspicion of illegal immigrants, [the] Metro Police [who execute the eviction] will contact the Department of Home Affairs’. The Allocation of Erven Within the Informal Settlement Upgrading Housing Programme (2010) cites its policy of ‘dealing with illegal immigrants residing within the informal settlement’ as a case for the DHA. The DHA is the government agency tasked with managing deportations, in line with its mandate as ‘custodian, protector and verifier of the identity and status of citizens and other persons resident in SA’.

## Discussion

Our analysis of how migration and health is presented in written policy documents of different levels of South African government highlights two key issues: 1) a void in the migration and health policy agenda of the South African government, evidenced by the absence of engagement with this issue in over half the policies reviewed in the health sector and other sectors that intersect with health, and; 2) where migration and health are considered (across the different sectors), the government’s engagement with the issue being both limited and largely negative. These findings have critical implications for advancing attainment of healthcare equity in SA regarding migrant and mobile groups.

The policy ‘absence’ regarding migration in relation to health and health-related issues signals the lack of political priority given to inclusive policy responses linked to migration. Policy-making is often defined as having a ‘problem–solution’ character: issues are framed by issue stakeholders in certain ways and the resulting narratives favour certain solutions and exclude others [51, 52]. Stakeholders often explicitly or implicitly mobilise certain ‘frames’ to shape perceptions of the legitimacy and appropriateness of their suggested policy responses [43]. Our finding that over half the policies identified across the health sector and other sectors relevant to health paid negligible or no attention to the issue, whilst those that did address it were vague, suggests that the South African government does not highly prioritise the issue of migration and its links to health. As has been argued from the review of global policy documents on migration [25], and relating to policymaking more generally [53, 54], what is emphasised or omitted from policy documents are often conscious tactical decisions that shape policy responses to human mobility. After all, policy making is inherently political and reflects stakeholders’ values and interests [55].

Across the policies that do take a position on the issue of migration (albeit weak), the language used and the ideas conveyed themselves present challenges for advancing policy engagement with issues of migration and health. Largely speaking, the discourse of the policy documents positions migration as problematic, including in the policies that do not explicitly refer to health, and discusses migration in terms of the various ‘threats’, as listed and described below.

Firstly, the dominant concern regarding migration in the health policies was the spread of disease, most notably communicable diseases. In national policies in particular, specific groups of cross-border migrants are seen as ‘vulnerable’ and at ‘high-risk’ of contracting and spreading HIV, TB and malaria. Health system efforts proposed to mitigate this ‘threat’ focus mainly on developing individual-level responses, most notably communication and health promotion strategies targeting these groups, rather than addressing the needs of migrants or the structural determinants of health. Likewise, none of the health policies engage with the process of migration and associated health systems challenges, in spite of the legislation affording non-citizens the right to health services [56]. The representation of cross-border migrants as vulnerable further serves to naturalise discriminatory attitudes and practices towards them. Indeed, we found that the narrative of cross-border migrants as threats to national security underlies multiple national and sub-national policies.

The second commonly identified theme in regard to migration, beyond the health sector and across the national and local levels of government, is about the threat of illegality and criminality linked to foreign nationals, with arguments presented for a risk-based security approach linked to stricter immigration and border securitisation. This likely reflects and also contributes to a xenophobic narrative in SA and reflects assertions of exclusive nationalism as a defence against cross-border migrants’ perceived threats [25]. The pattern of securitisation and reinforcement of border controls in response to cross-border migrants is common globally, and it undermines the health of migrants, including the development and implementation of appropriate public health responses to migration and population mobility [23]. Increased framing of migration as a security risk negatively affects health by impairing efforts to develop migration-aware and mobility-competent cross-border, regional health system responses. The implementation of corresponding policy would likely result in a growing number of irregular migrants, who due to fear of arrest, detention and deportation would avoid using health services for both prevention and treatment of disease, with negative effects for all [57, 58]. The public health community thus cautions that misapplying security approaches to justify nationalism and xenophobic attitudes, rather than protecting nations or their citizens, will likely result in the negative effects of failing to integrate migration and population mobility into health systems, presenting further challenges to making progress towards UHC goals [19].

In addition, the dominant focus on ‘borders’ in the vast majority of national level policies reviewed (in the health sector and beyond, as summarised in Table 2, with the only exception being the links presented between migration, urbanisation and NCDs), means overlooking the health implications of internal mobility, which accounts for the most significant type of migration in SA [25, 27]. In particular, the country has experienced a high rate of urbanisation, with almost 60% of the population estimated to be urban [45]. Further, evidence suggests increasing numbers of internal circular labour migrants becoming ill in the urban areas where they work and return home in times of sickness, placing the burden of care on the rural healthcare system, which is relatively under-resourced [59, 60]. However, this phenomenon is not acknowledged as an issue facing the health system in the national of provincial level discourse on migration and health. The only reference we found to internal circular labour migration across the policies reviewed was at the local level, pertaining to Buffalo city, in regard to the challenges of providing temporary housing for the city’s circular labour migrants.

Whereas in many of the sub-national policies in the health sector and beyond migration is discussed largely through the lens of internal mobility – and particularly in the context of inter-provincial migration and rural–urban migration – even here, the dominant perception is negative, with migrants seen as taking up space and resources. This third representation of migration, as threatening and burdening public (health) services and amenities, presents its own challenges to advancing the issue of migration and health. While there are undoubtedly high levels of vulnerability to adverse health outcomes among migrants in SA (both domestic and cross-border migrants), there are often similarly high levels of vulnerability among local populations. Evidence from four African cities (Johannesburg, Maputo, Nairobi and Lubumbashi) suggests that displaced people, for example, are not the most vulnerable of urban residents. That in some contexts non-migrant groups face similar if not greater levels of vulnerability has implications for policies based on the perception of greater disadvantage in non-nationals and/or newcomers [61].

In addition, representing migrants arriving in cities as dependents in need of housing and other assistance, and rarely as people capable of self-sustenance, adds to the sense of panic and concern often characterising SA’s perception of migrants [25]. In reality, local governments have a ‘development mandate’ that calls for municipalities to develop sustainable interventions to address the social, economic and material needs of their populations, and the challenges of urban growth, migration, informal settlements and HIV [45]. However, in our analysis, we identified only a handful of policies that recognise the role of the municipality government in ensuring that ‘newcomers’ and ‘visitors’ have access to services. Even these policies are themselves problematic as the language of inclusivity is ambiguous or exclusionary of certain migrant groups, including undocumented migrants, refugees and/or asylum seekers. Beyond the few policies advocating positive approaches to serve migrants, the vast majority of system responses identified at the local level focus on addressing resource burdens and the ‘strain on public (health) services’ posed by new arrivals. Such responses are harmful, supporting the claims described as conjured regularly by xenophobic politicians and fear mongers in SA of hordes flooding cities from rural areas and other distant lands to threaten prosperity [25]. Our findings are echoed in a case study of migration in SA; internal and domestic migrants in SA’s largest cities continue to be seen mainly as a drain on public resources, rather than as potential resources, or as people the government is dedicated to serve [62].

The fourth overarching narrative in the policy discourse, at the intersection of national and provincial levels, is about the in-migration of low skilled workers, juxtaposed with the out-migration of high skilled workers – with these phenomena viewed collectively as a threat to SA’s development. This language in the policy documents in regard to these issues denotes cross-border migrants into the high skilled ‘desirable’ versus low skilled ‘undesirable’ categories, with corresponding strategies that push for immigration policy that prioritises the procurement of skilled labour migration, including with regards to health care workers. The coveting of highly skilled migrants fuels the exclusion of low skilled labour migrants entering the country, with implications for their health and well-being. As noted elsewhere, there are no options for low-skilled economic migrants entering the country to regularise their stay, who rely on the asylum system as the only option to regularise their stay [63]. However, two key national frameworks that address asylum seekers’ entitlements have inconsistencies that highlight the disconnect in public policy on asylum seekers’ rights to services, including healthcare. The 2019 and most recent NHI Bill [35] denotes that an asylum seeker or ‘illegal foreigner’ is (only) entitled to emergency services and services for notifiable conditions of public health concern, such as HIV or TB. Meanwhile, the White Paper on International Migration (2017) explicitly excludes asylum seekers from certain services, including integration policies, because of their temporary status. Indeed, a recent study undertaken in SA that analysed national legal frameworks on migration, alongside interviews with health policy actors on UHC and the NHI, highlights both the lack of cohesive national frameworks on migrants and refugees, and a further disjuncture between international treaties, the South African Constitution and national legislation [57]. Further, the key stakeholder interviews from this study reveal that in practice, health access barriers are perpetuated by the failure of healthcare workers to implement legal service obligations to migrants and refugees, either due to ignorance or blatant discrimination.

This study aimed to examine how the issue of migration and health is engaged with and framed in the published policies of the SA government including the types of policy responses proposed. We recognise that policy making is a wider enterprise involving a range of actors, with transnational and local processes often diverging from government policy priorities and initiatives, and that policies can take the form of explicit written documentation as well as being more implicit and taking form through institutional rules and norms [64]. A more in-depth, nuanced understanding of the policy processes linked to migration and health in SA would involve examining these issues in policy documents outside the public policy domain and pertaining to a range of actor types, including those from non-governmental organisations, as well as international and more local actors – as well as engaging with both written and unwritten policy processes. Such research could reveal valuable insights into the role of additional policy actors in reifying or modifying policy stances adopted by the South African government.

Overall, our analysis of South African government policies across different sectors and levels of government points to the systematic and widespread exclusion of certain migrant groups, which renders them ‘high risk’ to adverse health outcomes. The term ‘healthy migrant effect’ (p.633) [27] is used to describe the observation that recent arrivals are generally in better health than the local population. However, in many contexts including in SA, this health benefit often deteriorates as a result of the challenges these recent arrivals face to their health, exacerbated by the social exclusion and socio-economic difficulties resulting from barriers to accessing healthcare and other services that affect health outcomes [27, 38]. However, we did not identify a single policy mentioning the government’s shortcomings in providing protection, or the weakness of the country’s refugee policy framework. Rather, the policy documentation we identified from the level of national government instead focuses on cross-border migrant groups in terms of the spread of disease and insecurity in the country, and by doing so it largely overlooks internal mobility, and so contributes to the precarity and ‘vulnerability’ facing some migrants [25].

## Conclusions

Our study highlights that although SA has committed to UHC goals, including the care of migrant and mobile groups, the issue of migration in terms of how it relates to health, and the wider social and structural determinants of health, is poorly recognised in the policy documentation of the government at multiple levels, and across sectors. When considered, the discussion is skewed towards the importance of procuring highly skilled international workers to meet development goals and address shortages in the health workforce, or on the potential negative aspects of migration. As with other studies, we identified harmful and unsupported assumptions relating to the prevalence of cross-border migration, the spread of diseases, the link between immigration and security risks, and the burden of migration on health systems [1, 27]. These positions undermine approaches that are migration-aware and mobility-competent and that would support migrants’ health and wellbeing, in line with UHC goals [27, 28].

We found that the coveting of highly skilled foreigners was juxtaposed against other migrant groups, who are perceived to be a ‘threat’ to the country. This lens through which migration is largely represented is an ideological device that reinforces a crisis-driven policy response to migration to address these ‘threats’[25]. Such a response attributes blame and privileges actions that exclude certain migrant groups, counter to the UHC principle of inclusivity. Rather than engaging with migration in a more balanced way that recognises both external and internal mobility, and the importance of addressing the health needs of all people, and the positive aspects and benefits to SA of population mobility, the current policy narrative fuels nationalist and anti-migrant sentiment, compounding the ability for constructive engagement with the issue. Indeed, although migration has been shown to bring economic benefits to the socio-economic development of both countries of origin and destination, the wider societal benefits of migration and health are often overlooked, particularly in the context of a resource-constrained health system, as is the case in SA [38].

We argue, based on our study of SA that, on their own, commitments to UHC and legislation supporting migrants’ rights to health care will not bring about the change needed if the issue of migration is poorly considered in the sectors responsible for developing appropriate health responses and/or if the existing discourse on migration is pervaded by negative and discriminatory attitudes to migrants. Other scholars in SA indicate that the wider societal attitudes to migration are often characterised by xenophobia and a negative view of what migrants contribute to society – a perception reinforced by the policies and practices of government and other institutions including the media [65, 66].

There is an urgent need in SA to address bureaucratic injustices towards migrants, which contribute to xenophobia and further bureaucratic injustices. Anti-immigrant views stall progress towards achieving UHC and stoke anti-immigrant sentiments that lead to violence. Improving engagement with the issues of migration and health in SA requires government policymakers and practitioners to actively consider migration across sectors and policies that affect health outcomes. Migration and health is a multi-sectoral issue and requires a “whole of government approach” to support effective responses, rather than isolated actions by different agencies and stakeholders (p.5) [67]. Importantly, this approach should entail a concerted effort to shift harmful political and societal attitudes to migration at the institutional level and beyond.

Moving forward, and drawing on the experiences of Sri Lanka [68], a review of existing evidence should be undertaken and gaps in knowledge identified to develop an informed, action-based research agenda. The development of this research agenda should involve governmental and non-governmental actors, and relate to establishing a national migration and health task team to develop a National Migration and Health Policy and Action Framework (see Vearey, Hui & Wickramage 2020 for further detail [16]). Economic studies that investigate the effect of restricted healthcare access to migrants may provide the necessary data to affect some change, particularly at the level of policy-making. For example, a study in Germany examined the effects of restricted healthcare access and found that the cost of exclusion was much higher [69]. However, these types of data-driven studies will not shape policy if not accompanied by advocacy at both political and societal levels to shift pervasive perceptions regarding xenophobia and racism, which ultimately undermine efforts towards healthcare equity for migrant and mobile groups. A 2021 study of health policy actors in regard to UHC and NHI in SA [57] points to health access barriers being perpetuated by the failure of health workers to implement legal service obligations to migrant and mobile groups due to discrimination. Behavioural change research from social psychology demonstrates the challenges to the uptake and implementation of laws when there is a disconnect between the legal norms (the formal explicit rules of behaviour commanded by the State) and the societal norms (the informal implicit rules governed by shared social perceptions) regarding acceptable behaviour [70]. For example, xenophobia can be a strong social norm practice in defiance of the legal norms that forbid discrimination. The migration and public health community should look to how best to create lasting shifts in people’s perception of migrants. In this light, the social norms literature provides insights gaining traction in recent years in the public health and development community on how to transform ‘sticky’ norms underlying discriminatory practices, including racism and xenophobia [71–73]. Learning from the politics of developing and delivering migration and health policies and practices in other LMIC country contexts will provide South Africa with insights into how to effectively navigate this complex and politically unpalatable terrain. This includes drawing from for example the current experiences of Thailand and Sri Lanka [16, 68, 74, 75] in implementing approaches to achieve UHC through inclusive policies that engage with migration and mobility.

## Supplementary Information

**Additional file 1. **Automated search strategy: South African National, Provincial and Metropolitan-level policies.

**Additional file 2.**

**Additional file 3. **List of policies included in analysis.

## Data Availability

All data generated or analysed during this study are included in this published article and its supplementary information files.

## Abbreviations

WHA: World Health Assembly
WHO: World Health Organisation
UHC: Universal Health Coverage
SA: South Africa
NHI: National Health Insurance
NCDs: Non-Communicable Diseases
NDoH: National Department of Health
HIV: Human Immunodeficiency Virus
TB: Tuberculosis
STIs: Sexually Transmitted Infections
DHA: Department of Home Affairs
DoH: Department of Health
