# Supplementary material for: An analysis of migration and implications for health in government policy of South Africa
Source: Int J Equity Health. 2023 May 8;22:82. doi: 10.1186/s12939-023-01862-1 (PMC10165765; doi:10.1186/s12939-023-01862-1)
Supplement: Supplementary file 1 — Additional file 1. Automated search strategy: South African National, Provincial and Metropolitan-level policies. [file 12939_2023_1862_MOESM1_ESM.docx]

| Additional filesAdditional file 1 Automated search strategy: South African National, Provincial and Metropolitan-level policies | | | |
| --- | --- | --- | --- |
|  | **Database Searched** | **Search Terms** | **Limits** |
| National | ***Peer-Reviewed Literature***  Scopus and Google Scholar | Migration/migrat*/migrant*/ AND South Afric* AND policie*/policy | - 2007/8-onwards - English - Only national-level policies |
|  |  | Migra* AND health AND national AND South Afric* and policie*/policy |  |
|  |  | National department of health AND health AND policie*/policy AND South Africa* |  |
|  |  | *health AND *South Afric* AND National department of health AND policie*/policy |  |
|  |  |  |  |
|  | ***Gray Literature*** |  |  |
|  | NDOH-website, NDOH Data Dictionary, Google, UCT Libraries and other | Combination of above terms with strategic report*/plan*/annual performance plan*/report*/financial report*/quarterly report* |  |
|  |  |  |  |
| Provincial | ***Peer-Reviewed Literature***  Scopus and Google Scholar | Migration/migrat*/migrant*/ AND South Afric* AND policie*/policy AND provinc* (Use of each province name for each search) | - 2007/8-onwards - English - Only provincial-level policies |
|  |  | Migra* AND health AND national AND South Afric* AND policie*/policy AND (name of province searched) |  |
|  |  | Health AND policie*/policy AND (name of province searched) |  |
|  |  |  |  |
|  | ***Gray Literature*** |  |  |
|  | NDOH-website, NDOH Data Dictionary, Google, UCT Libraries and other | Combination of above terms with strategic report*/plan*/annual performance plan*/report*/financial report*/quarterly report* |  |
|  |  |  |  |
| Metropolitan | ***Peer-Reviewed Literature***  Scopus and Google Scholar | Migration/migrat*/migrant*/ AND South Afric* AND policie*/policy AND provinc* (Use of each metropolitan municipality name for each search) | - 2002-onwards; - English - Only municipal-level policies |
|  |  | Migra* AND health AND national AND South Afric* AND policie*/policy AND (name of metropolitan municipality searched) |  |
|  |  | Health AND policie*/policy AND (name of metropolitan municipality searched) |  |
|  | ***Gray Literature*** |  |  |
|  | NDOH-website, NDOH Data Dictionary, Google, UCT Libraries and other | Combination of above terms with strategic report*/plan*/annual performance plan*/report*/financial report*/quarterly report* |  |
