# Supplementary material for: An analysis of migration and implications for health in government policy of South Africa
Source: Int J Equity Health. 2023 May 8;22:82. doi: 10.1186/s12939-023-01862-1 (PMC10165765; doi:10.1186/s12939-023-01862-1)
Supplement: Supplementary file 2 — Additional file 2. [file 12939_2023_1862_MOESM2_ESM.docx]

### Additional file 2

The additional file 2 includes all the policy documents reviewed at national and sub-national levels in SA (227 in total), including (where relevant) the data extracted from each document, as follows:

1. National Department of Health and Provincial Department of Health policies:

[Additional file 2a.xlsx](https://docs.google.com/spreadsheets/d/1AgrbxvrYb_D4BPmeLGwqB4F4mAHIw3OH/edit#gid=652821179)

1. Policies in other Departments:

[Additional file 2b.xlsx](https://docs.google.com/spreadsheets/d/1JTYpXVpP0VHNSQH6kAafec8joTjQE64B/edit?usp=drive_web&ouid=112913617526893694818&rtpof=true)

1. Metropolitan Municipality policies:

[Additional file 2c.xlsx](https://docs.google.com/spreadsheets/d/1pQuhbD0b2MKBtr5Vj4xasu1O5ONdouav/edit?usp=drive_web&ouid=112913617526893694818&rtpof=true)
