# Supplementary material for: An analysis of migration and implications for health in government policy of South Africa
Source: Int J Equity Health. 2023 May 8;22:82. doi: 10.1186/s12939-023-01862-1 (PMC10165765; doi:10.1186/s12939-023-01862-1)
Supplement: Supplementary file 3 — Additional file 3. List of policies included in analysis. [file 12939_2023_1862_MOESM3_ESM.docx]

### Additional file 3

**List of policies included in analysis**

| **Policy** | **Year** |
| --- | --- |
| **National level (34 policies)** | |
| 1. (DoH) Strategic Priorities for the National Health System | 2004-2009 |
| 1. (DoH) Employment of Foreign Health Professional in the South African Health Sector | 2010 |
| 1. (DoH) Tuberculosis Strategic Plan for South Africa | 2007-2011 |
| 1. (DoH) Cervical Cancer Prevention and Control Policy | 2017 |
| 1. (DoH) Breast Cancer Prevention and Control Policy | 2017 |
| 1. (DoH) Malaria Elimination Strategic Plan | 2012-2018 |
| 1. (DoH) Policy Guideline on the Requirements for Practice of Medical Professionals in South Africa | 2018 |
| 1. (DoH) South African Guidelines for the Prevention of Malaria | 2018 update |
| 1. (DoH) Strategy for the Prevention and Control of Obesity in South Africa | 2015-2020 |
| 1. (DoH) South Africa’s National Strategic Plan for HIV, TB and STIs | 2017-2022 |
| 1. (DoH) Malaria Elimination Strategic Plan for South Africa | 2019-2023 |
| 1. (Department of Environmental Affairs) National Framework for Sustainable Development in South Africa | 2008 |
| 1. (Office of the Presidency) Joint Initiative on Priority Skills Acquisition | 2008 |
| 1. (Department of Social Development) Integrated Social Crime Prevention Strategy | 2011 |
| 1. (DHA) Department of Home Affairs Strategic Plan | 2009/2010-2011/2012 |
| 1. (Department of International Relations and Cooperation) Strategic Plan | 2009-2012 |
| 1. (Department of Justice and Constitutional Development) Strategic Plan Annual Review | 2011-2012 |
| 1. Review: (Department of Arts and Culture): A National Strategy for Developing an Inclusive and Cohesive South African Society | 2012 |
| 1. (Department of Tourism) Rural Tourism Strategy | 2012 |
| 1. (Department of International Relations and Cooperation) Strategic Plan | 2010-2013 |
| 1. (Department of Education) Human Resource Development Strategy for South Africa | 2010-2013 |
| 1. (Department of Home Affairs) Strategic Plan | 2010/11-2012/13 |
| 1. (Department of Defence) Safeguarding South Africa For a Better Life for All: Strategic Plan | 2010/11-2012/2013 |
| 1. (Department of Economic Development) Economic Development Medium Term Strategic Plan | 2010/2011-2102/2013 |
| 1. (Office of the Presidency) Together Doing More and Better: Medium-term Strategic Framework | 2009-2014 |
| 1. South African Police Service Strategic Plan | 2010-2014 |
| 1. (Department of Social Development) Strategic Plan | 2010-2015 |
| 1. (Department of Social Development) Strategic Plan | 2012-2015 |
| 1. (Department of Labour) Strategic Plan | 2011-2016 |
| 1. (Department of Labour) Strategic Plan | 2012-2017 |
| 1. White paper on International Migration | 2017 |
| 1. (Department of Social Development) Strategy and Guidelines for Children Living and Working in the Streets | Undefined |
| 1. (Office of the Presidency) Our Future Make It Work: National Development Plan 2030 | Undefined |
| 1. (Department of Economic Development) The New Growth Path: Framework | Undefined |
| **Provincial level (33 policies)** | |
| 1. (Planning Commission) North West Development Plan | 2013 |
| 1. (DoH) KwaZulu-Natal Annual Performance Plan | 2012-13 |
| 1. (DoH) Western Cape Youth Development Strategy | 2013 |
| 1. (DoH) Gauteng Annual Report | 2012-2014 |
| 1. (DoH) Healthcare 2030: The Road to Wellness | 2014 |
| 1. (DoH) Free State 5- Year Strategic Plan | 2010/11- 2014/15 |
| 1. (DoH) Limpopo Provincial Government Department of Health Annual Report | 2014-15 |
| 1. (DoH) Eastern Cape Annual Report | 2014-2015 |
| 1. (DoH) Limpopo Annual Report | 2014-2015 |
| 1. (DoH) Mpumalanga Annual Report | 2014-2015 |
| 1. (DoH) KwaZulu-Natal Annual Performance Plan | 2013/14-2015/16 |
| 1. (DoH) Eastern Cape Annual report | 2015-2016 |
| 1. (DoH) Mpumalanga Annual Report | 2016-2017 |
| 1. (DoH) North-West Department of Health Annual Report | 2016-2017 |
| 1. (DoH) Gauteng Department of Health Annual Report | 2017-2018 |
| 1. (DoH) KwaZulu-Natal Annual Report | 2017-2018 |
| 1. (DoH) Mpumalanga Annual Report | 2017-2018 |
| 1. (DoH) Limpopo Provincial Government Department of Health Annual Report | 2017-2018 |
| 1. (DoH) Northern Cape Annual Performance Plan 2018 | 2017-2018 |
| 1. (DoH) Eastern Cape Annual Health Performance Plan | 2017-2018 |
| 1. (DoH) Western Cape Government Provincial Strategic Plan | 2014-2019 |
| 1. (DoH) KwaZulu-Natal Department of Health Annual Performance Plan | 2016/17-2018/19 |
| 1. (DoH) Mpumalanga Annual Report | 2018-2019 |
| 1. (DoH) Mpumalanga Annual Performance Plan | 2018-2019 |
| 1. (DoH) Gauteng Department of Health Annual Report | 2017-2018 |
| 1. (DoH) Gauteng Department of Health Annual Report | 2018-2019 |
| 1. (DoH) Eastern Cape Annual Health Performance Plan | 2018-2019 |
| 1. (DoH) Eastern Cape Department of Health Strategic Plan | 2015/16-2019/20 |
| 1. (DoH) KwaZulu-Natal Department of Health Annual Performance Plan | 2017/18-2019/20 |
| 1. (DoH) Northern Cape Annual Performance Plan | 2017/18-2019/2020 |
| 1. (DoH) Eastern Cape Annual Health Performance Plan | 2019-2020 |
| 1. (DoH) Gauteng Health Council Strategic Implementation Plan | 2017-2022 |
| 1. (DoH) Free State Department of Health Annual Performance Plan | 2019/20-2021/22 |
| **Metropolitan level (34 policies)** | |
| 1. Ekurhuleni Agricultural Development Policy | 2002 |
| 1. Ekurhuleni Housing Assistance in Urgent Housing Situations Policy | 2003 |
| 1. Provision of Road Tolls in the City of Cape Town | 2004 |
| 1. Ekurhuleni Procedure for the Removal of Unlawful Occupiers of Land | 2005 |
| 1. Tshwane Annual Report | 2008-2009 |
| 1. Ekurhuleni Policy on HIV & AIDS at the Workplace | 2009 |
| 1. Nelson Mandela Bay Metropolitan Spatial Development Framework | 2009 |
| 1. Tshwane Annual Report | 2009-2010 |
| 1. Allocation of Erven Within the Informal Settlement Upgrading Housing Programme | 2010 |
| 1. Buffalo City Annual Report | 2011-2012 |
| 1. Tshwane Annual Report | 2011-2012 |
| 1. Johannesburg Disaster Management Plan | 2012 |
| 1. Ekurhuleni Youth Policy | 2012 |
| 1. Buffalo City Annual Report | 2013-2014 |
| 1. Ekurhuleni Environmental Policy and Implementation Plan | 2013 |
| 1. Cape Town Vulnerable Groups Policy | 2013 |
| 1. Consolidated Annual Report for Tshwane | 2012-2014 |
| 1. Buffalo City Annual Report | 2013-2014 |
| 1. Johannesburg Spatial Development Framework 2040 | 2014-2015 |
| 1. The Cape Town Bioregional Plan | 2015 |
| 1. eThekwini Integrated Development Plan | 2015-2016 |
| 1. eThekwini Integrated Development Plan | 2016-2017 |
| 1. Cape Town Climate Change Policy | 2017 |
| 1. Mangaung Annual Report | 2017-2018 |
| 1. Mangaung Consolidated Annual Report | 2018-2019 |
| 1. Tshwane Annual Report | 2018-2019 |
| 1. eThekwini Integrated Development Plan | 2019-2020 |
| 1. Mangaung Integrated Development Plan | 2019-2020 |
| 1. Mangaung Metropolitan Spatial Development Framework | 2020 |
| 1. Mangaung Metropolitan Municipality Five Year Integrated Human Settlements Plan | 2016/17-2020/21 |
| 1. eThekwini Integrated Development Plan | 2020-2021 |
| 1. eThekwini Integrated Development Plan | 2017/18-2021/22 |
| 1. Nelson Mandela Bay Integrated Development Plan | 2017/18-2021/22 |
| 1. Nelson Mandela Bay Long-term Growth and Development Plan | 2017-2032 |
